# Alternative RNA splicing in cancer: what about adult T-cell leukemia?

**DOI:** 10.3389/fimmu.2022.959382

**Published:** 2022-08-01

**Authors:** Julie Tram, Jean-Michel Mesnard, Jean-Marie Peloponese

**Affiliations:** Institut de Recherche en Infectiologie de Montpellier (IRIM), Centre national de la recherche scientifique (CNRS), Université de Montpellier, Montpellier, France

**Keywords:** HTLV-1, Alternative splicing, Oncogenesis, Leukemia, Chemoresistance

## Abstract

Eukaryotic cells employ a broad range of mechanisms to regulate gene expression. Among others, mRNA alternative splicing is a key process. It consists of introns removal from an immature mRNA (pre-mRNA) *via* a transesterification reaction to create a mature mRNA molecule. Large-scale genomic studies have shown that in the human genome, almost 95% of protein-encoding genes go through alternative splicing and produce transcripts with different exons combinations (and sometimes retained introns), thus increasing the proteome diversity. Considering the importance of RNA regulation in cellular proliferation, survival, and differentiation, alterations in the alternative splicing pathway have been linked to several human cancers, including adult T-cell leukemia/lymphoma (ATL). ATL is an aggressive and fatal malignancy caused by the Human T-cell leukemia virus type 1 (HTLV-1). HTLV-1 genome encodes for two oncoproteins: Tax and HBZ, both playing significant roles in the transformation of infected cells and ATL onset. Here, we review current knowledge on alternative splicing and its link to cancers and reflect on how dysregulation of this pathway could participate in HTLV-1-induced cellular transformation and adult T-cell leukemia/lymphoma development.

## Introduction

Cancer burden remains today’s second worldwide leading cause of mortality, with up to 10 million deaths reported in 2020 (1). Among other known risk factors, a substantial part of almost 15% of cancers is directly linked to infectious agents, especially viruses (2–4). To date, seven viruses have been described as oncogenic for humans, among which are DNA viruses: Human Papillomavirus (HPV), Epstein-Barr virus (EBV), Kaposi’s sarcoma-associated herpesvirus (KSHV), Hepatitis B virus (HBV) and Merkel cell polyomavirus (MCPyV); one RNA virus: Hepatitis C virus (HCV); and one retrovirus: Human T-cell leukemia/lymphoma virus type 1 (HTLV-1) (3, 5). HTLV-1 is a member of the *Retroviridae* family and was first discovered in the early ‘80s (6, 7). Later on, it has been linked to a rare cancer named adult T-cell leukemia/lymphoma (ATL) (8, 9) as well as to the HTLV-1 associated myelopathy or tropical spastic paraparesis (HAM/TSP) inflammatory disease (10, 11). HTLV-1 infects roughly 10 million people in localized endemic clusters, the main ones being Southwest Japan, the Caribbean area, Central and South America, and West Africa (12). Most HTLV-1 infected individuals are asymptomatic, but 5% of the latently infected are at risk of developing ATL (13, 14) and another 5% of HAM/TSP (11).

## HTLV-1 infection and oncogenes

**Figure 1:**
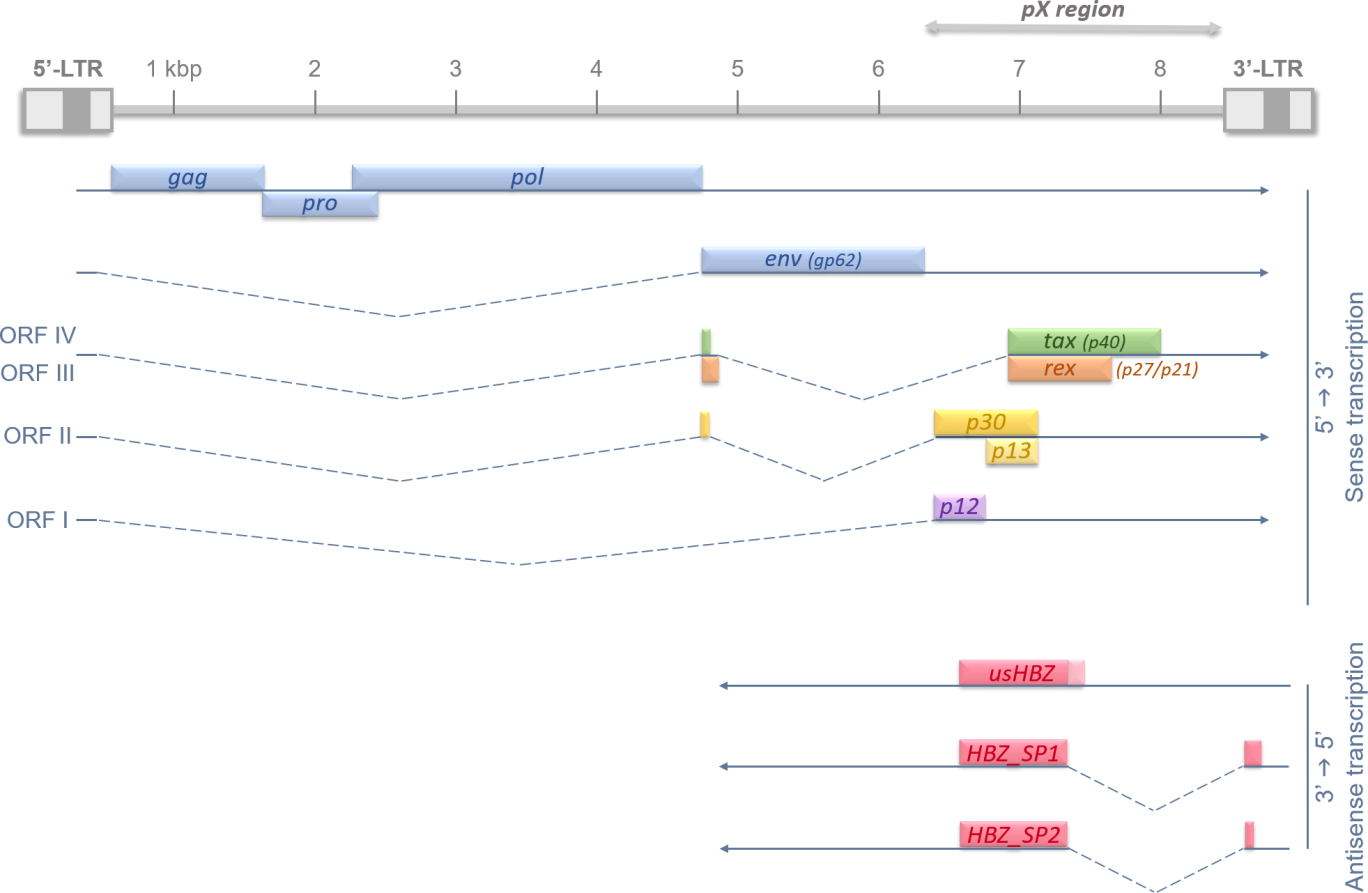
Recapitulative diagram of HTLV-1 proviral genome, Open Reading Frames (ORFs), and splicing-regulated genes. HTLV-1 genome is 9 kb long and flanked on each side by 5’ and 3’ Long Terminal Repeats (LTRs). HTLV-1 LTRs are approximately 750 bp in length and are segmented in U3, R and U5 regions (in order). U3 region usually contains enhancer and promoter sequences which drive viral transcription; and R domain encodes the 5′ capping sequences (5′ cap) and the polyadenylation (pA) signal. The structural genes gag, pro, pol and env are encoded in the 5’ part of the provirus, and regulatory and auxiliary proteins are encoded in the pX region, at the 3’ side. The transcription initiates in the promoter-bearing 5’-LTR in 5’→3’ for all viral genes, except for the antisense transcripts of hbz. hbz is encoded by the proviral complementary strand and its transcription, initiated in the 3’-LTR, occurs in an antisense fashion (3’→5’). The different Open Reading Frames (ORFs) are indicated in roman numbers and splicing events are shown by dotted lines.

Even though HTLV-1 can infect multiple types of immune cells (lymphocytes, dendritic cells, macrophages…), the main targets remain the CD4^+^ effector/memory T lymphocytes (15). HTLV-1 9kb-long proviral genome is composed of a positive-sense single-stranded RNA molecule, framed by two Long Terminal Repeat (LTR) regions in 5’ and 3’ (16), and randomly integrates into the host cell genome (17, 18). Of note, retroviral LTRs are known to harbor bi-directional promoters (19). The neosynthesized viral particles can spread *via* a cell-cell contact through a viral synapse (20) but it is the clonal expansion of infected cells that is responsible for the proviral burden (21). HTLV-1 genome contains several regions encoding for either structural proteins such as Gag (structural protein), Pro (protease), Pol (reverse transcriptase), and Env (envelope protein), or auxiliary and regulatory proteins like Tax and Rex. All the latter genes are expressed through transcription initiation in the promoter harbored by the 5’-LTR (22). However, a major study published in 2002 described a tenth protein-encoding gene with a transcription initiating in the 3’-LTR (23). This antisense transcript and protein is named HBZ for HTLV-1 Basic leucine Zipper (bZIP) transcription factor. HTLV-1 genome organization was summarized in **Figure 1**. So far, many studies have proven the crucial role of both Tax and HBZ in HTLV-1-associated persistence, pathogenesis, cellular transformation, and ATL development (24, 25).

### Tax

Tax was initially described as the viral transactivation protein of HTLV-1 because of its capacity to promote 5’-LTR transcription (26). Thus, it is an essential pawn in viral replication and *de novo* infection. Previous *in vivo* studies have also shown T-cell leukemia or lymphoma induction in transgenic mice when Tax alone is expressed (27) or simultaneously with HBZ (28). By modulating various cellular pathways, such as the NF-KB (29) or AP-1 (30) pathways, implicated in cell proliferation, apoptosis, and genomic stability, Tax plays a key role in cellular transformation and leukemogenesis onset (22). However, because it is a major antigen targeted by cytotoxic T lymphocytes, its expression is very often silenced in ATL cells. Indeed, nonsense mutations of the *tax* gene (31) and deletion and/or hyper-methylation of the 5’-LTR (32) explain why so few ATL patients display a stable Tax expression. Overall, HTLV-1 transactivator Tax remains necessary for the early steps of infection and cellular transformation onset, but not for their maintenance over time. Nonetheless, minor and sporadic expression of Tax is sufficient to trigger the antiapoptotic machinery. This mechanism once stimulated, continues even after Tax expression is switched off again, and allows the infection persistence (33). In other words, Tax transient silencing allows HTLV-1 stably infected cells to evade the immune system detection and to clonally expand.

### HBZ

Unlike Tax, HTLV-1 antisense protein HBZ is expressed in all ATL cells. Over the years, diversified techniques were used to confirm both Tax silencing and HBZ strong expression in ATL cells isolated from leukemic patients (34–36). Most recently, Matsuo et al. described a new intragenic enhancer region near the 3’-LTR that actively promotes and maintains antisense transcription, further confirming HBZ persistence over time (37). After its discovery in 2002 (23), another study manage to describe different transcripts, hence different potential protein isoforms of HBZ (**Figure 1**). Indeed, three transcripts were characterized in different T cell lines: one unspliced isoform usHBZ and two spliced HBZ_SP1 and HBZ_SP2. However, only usHBZ and HBZ_SP1 can be detected at the protein level in ATL patients’ PBMCs, HBZ_SP1 being the isoform with the strongest expression (38, 39). Like Tax, HBZ plays a crucial role in HTLV-1 pathogenesis and ATL development as both mRNA and protein expression of HBZ were shown to induce T cell proliferation; and the knockdown of *hbz* gene leads to cell death in infected/ATL cell lines (35, 40). Because retroviral infection/leukemic environment is so complex and HBZ functions so pleiotropic, it is quite difficult to have a global view of HBZ’s particular role in oncogenesis. *In vivo* experiments using HBZ-expressing transgenic mice have previously shown induction of the chemokine receptor CCR4 which resulted in a promotion of T-cell proliferation and migration, both needed for leukemogenesis (41). Moreover, our group showed that *hbz* gene expression leads to an increase in the AP-1 transcription factor JunD expression that correlates with the appearance of transformed cell features (42). We also described how HBZ induces the expression of a N-terminal truncated ΔJunD isoform. This isoform is unresponsive to the tumor suppressor *menin*, thus leading to cell proliferation and transformation (43). In addition, HBZ is responsible for the disruption of the microRNAs network leading to DNA-strand breaks and general genetic instability (44, 45). Barbeau and Mesnard previously thoroughly reviewed the different ways HBZ uses to down-regulate the viral sense transcription, meaning Tax expression, to help evade host immune surveillance. This down-regulation occurs simultaneously with antisense transcription promotion, hence its own, to maintain and amplify its effects (46). Taken together, those data give us an overview on the central role, among many other cellular pathways (25), HBZ plays in the oncogenic process leading to ATL.

## RNA splicing and cancer

**Figure 2:**
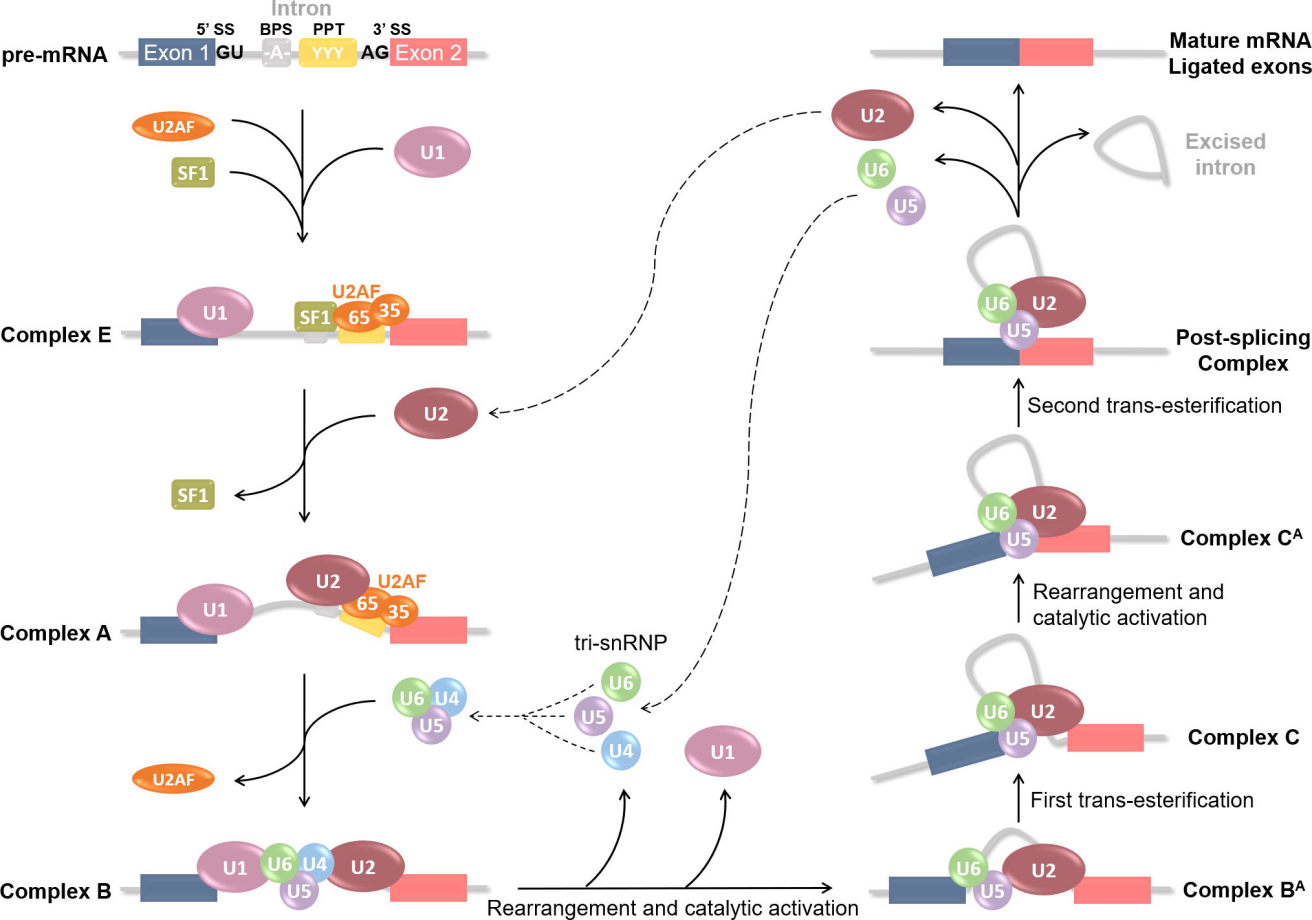
Step-by-step spliceosome assembly and pre-mRNA splicing reaction. Splicing is catalyzed by a large protein complex called the spliceosome. Spliceosome assembly requires a series of steps and intermediate complexes, and starts at the transcription site. It involves 5 small nuclear ribonucleoprotein particles (snRNPs): U1, U2, U4, U5 and U6; combined with roughly 300 associated proteins. Splicing is based on the recognition of 5’SS and 3’SS (splicing sites), also known as donor or acceptor sites, located at each end of an intron. Several cis-acting regulatory sequences are necessary such as the branching point sequence (BPS) and the poly-pyrimidine tract (PPT). Splicing begins with U1 snRNP recognition of the 5’SS and binding onto the pre-mRNA. U2 auxiliary factor (U2AF) 65 and 35kDa sub-units then respectively bind the PPT and the 3’SS; and Splicing Factor 1 (SF1) the BPS. These first steps form the E(arly) complex, which converts into pre-spliceosome **complex A** after U2 snRNP recruitment at the BPS and SF1 replacement. U2AF then leaves and U4, U5 and U6 pre-assemble into the tri-snRNP which is recruited to compose the pre-catalytic **complex B.** Rearrangement and catalytic activation into **complex B^A^** occur *via* U1 and U4 release. A first trans-esterification reaction is catalyzed and leads to the **Complex C** containing free Exon 1 and Intron-Exon 2 fragment. The complex undergoes additional rearrangement and activation, and the **Complex C^A^** catalyzes the second trans-esterification reaction to free Exon 2. Both exons are ligated in the Post-splicing Complex; U2, U5 and U6 as well as excised intron are released; and mature mRNA is formed. All snRNPs are recycled for additional rounds of splicing.

**Figure 3:**
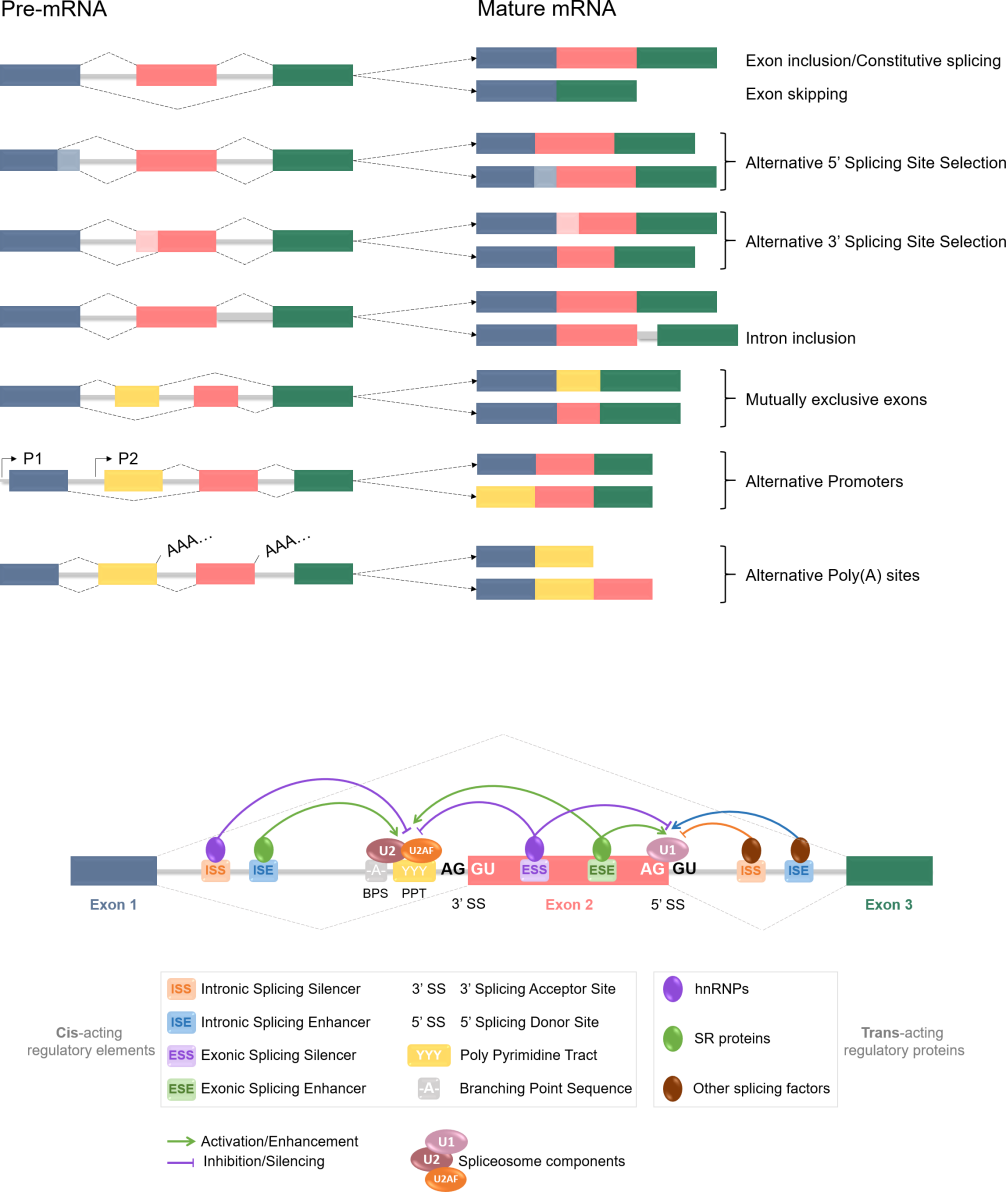
pre-mRNA alternative splicing events and regulation mechanisms. **(A)** Common constitutive and alternative splicing events are listed here. Colored boxes represent different exons, grey lines stand for introns, and dotted lines are splicing events. Exons are usually included or excluded (skipped) individually, but mutually exclusive exons involve the preferential retention of one exon at the expense of one or more others. Alternative 5’ and 3’SS selection induce exons modifications, as parts of the exons can be excluded during the process. Introns are mostly removed from the mRNA but inclusion can occur and often leads to nonsense-mediated decay or shift in the Open Reading Frame (ORF). Alternative promoters and poly **(A)** sites selection can also happen at the splicing level. All those events contribute to the increase of the proteome diversity. **(B)** Alternative splicing is regulated by trans-acting regulatory proteins, named splicing factors (SFs), binding to cis-acting regulatory sequences. SFs such as SR proteins and hnRNPs are RNA-binding proteins and are considered as enhancers and silencers respectively, since SR proteins are typically recruited to ISEs and ESEs while hnRNPs usually bind to ISSs and ESSs. However, increasing number of studies have revealed more context-dependent regulation roles for each. Other SFs are also at stake, and it is the balance and interplay between those splicing activators and repressors, and with the spliceosome components, that determines the splicing donor and acceptor sites selection for a splicing event to occur.

As mentioned before, RNA splicing is a key mechanism for gene expression regulation, allowing one gene to generate several distinct mature mRNAs. According to large-scale genomic studies, approximately 95% of human protein-encoding genes are subjected to alternative splicing (47), hence greatly increasing the cellular transcriptome and the proteome diversity. Considering the importance of RNA regulation in multiple cellular pathways such as differentiation, proliferation, survival, and death, any aberration in the splicing process could lead to various diseases occurrence. Alternative splicing is a highly regulated mechanism involving a massive protein complex called the spliceosome. Spliceosomal assembly requires a series of steps and intermediate complexes and starts at the transcription site (**Figure 2**). The spliceosome is composed of five small nuclear ribonucleoprotein particles (snRNPs): U1, U2, U4, U5, and U6; combined with roughly 300 associated proteins, and catalyzes pre-mRNA splicing reactions (48, 49). In short, it recognizes the splicing donor and acceptor sites and takes care of removing introns (non-coding sequences) to ligate exons together. A more detailed explanation of the splicing process can be found in **Figure 2**. Different kinds of splicing events can occur (listed in **Figure 3A**), therefore broadening the number of potential mature mRNAs and with that, protein isoforms, from a single coding gene. Alternative splicing is regulated by trans-acting regulatory proteins, also called splicing factors (SFs), binding to cis-acting regulatory sequences. SFs such as SR (serine/arginine-rich) proteins and hnRNPs (heterogeneous nuclear ribonucleoproteins) are RNA-binding proteins and are considered as enhancers (50, 51) and silencers (52, 53) respectively since SR proteins are typically recruited to ISEs and ESEs (respectively intronic & exonic splicing enhancers) while hnRNPs usually bind to ISSs and ESSs (**Figure 3B**). Disruptions of the splicing mechanism and regulation have been documented in many pathologies, ranging from genetic (54–56) and autoimmune (57, 58) diseases, to cancers; the latter being the center of discussion in the following sections.

### Splicing alterations in cancer onset and development

Alternative splicing is an efficient and key mechanism for proteomic variety. Hence, cancer cells could divert and take advantage of this process to produce aberrant proteins with additional, deleted, or modified biological functions, thus contributing to oncogenesis. We discuss here at what level pre-mRNA splicing can be altered and the implication of this misregulation in cancer onset and/or development. The first alteration level would be mutations in the splicing core machinery, namely the spliceosome components. This aspect, even if it seems obvious, has not been extensively studied in cancer biology, as mutations in the spliceosomal assemble often lead to cell death. Using whole-genome sequencing, Yoshida et al. revealed frequent mutations for at least six spliceosome-related proteins-encoding genes in myeloid neoplasm samples (59). Among others, U2AF35, a key factor in the early steps of spliceosome assembly (**Figure 2**) is redundant. Its mutations induce defects in 3’SS recognition and lead to major intron retention and with that, the introduction of termination codons in the mRNA, conducting to mRNA decay. Besides, mutations also significantly affected SF3B1, a component of U2 snNRP, in myeloid neoplasms but also in uveal melanomas, which it was more thoroughly studied (60, 61). Globally, alterations of any splicing machinery component are linked to cell death rather than growth (59, 62); however, they are still related to hematological disorders such as acute myeloid leukemia (59, 63).

The second level of RNA splicing alterations resides in mutations, or at least differential expression, of the trans-acting regulatory factors mentioned before (**Figure 3B**). This particular aspect has been extensively assessed in many cancers already, with a focus on the two main families of splicing factors: SR and hnRNP proteins. As an example, SRSF1 (SF2/ASF) is particularly overexpressed in diverse human tumors such as breast (64, 65) or lung cancers (64, 66). Its expression both *in vitro* and *in vivo* allows cells to proliferate and transform; and its deletion leads to a reversal effect (64), making it a defined proto-oncogene. Other SR proteins like SRSF3 (SRp20) and SRSF6 have been classified as proto-oncogenes as well, mostly in lung and colon cancers (67–69). Regarding the hnRNP family of proteins, recent genome-wide and pan-cancer omics data reveal that global overexpression of hnRNPs is linked with poor cancer prognosis. hnRNP A1, for instance, is highly expressed in most cancers (70) but seems to be a key proto-oncogene in particular in lung cancers where it antagonizes SRSF1 functions and deregulates the global RNA splicing environment (71, 72). We won’t be further detailing the links between splicing factors alterations and oncogenesis, as current knowledge has been broadly documented by others (73–75). Overall, even if SFs can act as oncogenes, it is the interplay between them and their expression balance that will determine the outcome, meaning the carcinogenic potential of the cells or not (71, 76).

Finally, the last level of splicing dysregulation we wanted to mention is regarding known cellular oncogenes and/or newly oncogenic splice variants. Because RNA splicing can produce protein isoforms with modified functions, alteration in this mechanism could lead to new isoforms, with potentially higher transformation properties. Hence, cancer-related protein variants could confer survival advantages to cells and be preferentially selected. A good example we can highlight is p53. p53 is known as a master regulator of many cellular pathways ranging from cell-cycle control, DNA repair, and apoptosis to immunity (77, 78). It has been shown that p53 is regulated by splicing as several protein isoforms have been described, associated with different cell fate regulation (79–81). p53 is generally called and considered a tumor suppressor for its DNA repair and cell-cycle controlling activities, however, in a cancer-related matter, p53 displays a differential expression of its isoforms (82–84), and a particular one is often mentioned: Δ133p53. This variant is promoting tumor progression and angiogenesis in mice models and escapes cellular senescence; making it a potential prognosis marker (79, 85, 86). This example illustrates the infinite possibilities of RNA splicing misregulation of key cellular genes in cancer development.

Taken together, these samples of explanation give us insights into how alternative splicing is altered at different levels in cancers. Adding to the complexity, this mechanism has also been described as implicated in various chemoresistance mechanisms (87–89) (not detailed here). Therefore, RNA splicing modulation is, without a doubt, a crucial element to consider in the development of new therapeutic strategies to prevent, ameliorate treatment efficiency, and fight cancer. In the remaining sections, we approach the peculiar matter of viral oncogenes and especially those of HTLV-1.

### Focus on viral oncogenes

Oncogenic viruses are yet another particular case. As mentioned before, there are seven known viruses capable of inducing cancer in humans; each of them expressing its own viral oncogenes (90–92). As the splicing mechanism is greatly impaired in many cancers, knowing if and how those viral genes are regulated at the splicing level seemed relevant in order to assess their global oncogenic properties. Indeed, splicing promotes proteome diversity by producing protein isoforms with potentially different biological functions. Hence, regulation of viral oncogenes by splicing might be critical for their associated pathogenesis and oncogenesis. We tried to review here whether the said viral oncogenes’ expression is regulated by alternative splicing or not, and if it is the case, what kind of alternative splicing events (ASEs) occur (**Table 1**). Surprisingly, splicing of those viral oncogenes is globally well documented, except for Hepatitis B and C viruses for which we did not find clear information in the literature. Then, we went even further by looking at whether or not the same viral oncogenes were known splicing regulators. Overall, most of the mentioned oncogenes’ expression is indeed regulated *via* different ASEs, and some are even known to alter the splicing mechanism themselves. This knowledge corroborates the current thinking about the link between RNA splicing dysregulation and pathogenesis, especially oncogenesis, as virally-induced cancers are no exception. In the last section, we focus on HTLV-1 particular case and review current insights on splicing regulation in HTLV-1 infection, focusing on its two oncogenes *tax* and *hbz*.

**Table 1:** Recapitulative table of human oncogenic viruses, associated cancers and alternative splicing regulation.

	Associated cancers	Oncogenes	ASEs implicated	Splicing modulation?
***Human DNA Viruses***				
Human Papillomavirus (HPV-16, 18, 31, 45)	Cervical, anal, vulvar, vaginal, penile, head and neck, skin cancer	E6, E7	Alternative 3’ SS Alternative Poly(A) Sites (93–95)	Yes (96–98)
Epstein-Barr Virus (EBV/HHV-4)	B and T cell, Burkitt’s and Hodgkin’s lymphomas; nasopharyngeal and gastric cancers	LMP-1, BARF-1	Alternative Promoters (91, 99, 100)	Implied (101–103)
Kaposi’s Sarcoma Herpesvirus (KSHV/HHV-8)	Kaposi’s sarcoma, pleural effusion lymphoma	LANA, vCyclin, vFLIP, vIRF2-3, LAMP, vGPCR	Alternative Promoters, 3’SS and Poly(A) sites (91, 104–106)	Implied (91, 107)
Hepatitis B Virus (HBV)	Hepatocellular carcinoma	HBx	/*(Alternative translation initiation)*	Yes (108, 109)
Merkel Cell Polyomavirus (MCV/MCPyV)	Merkel Cell Carcinoma	T antigens (large/small)	Alternative 5’ SS Intron retention (110–112)	N/D
***Human RNA Viruses***				
Hepatitis C Virus (HCV)	Hepatocellular carcinoma	Core protein, NS3, NS5A, and NS5B	N/D	Implied (108)
***Human Retroviruses***				
Human T-cell Leukemia/Lymphoma Virus Type 1 (HTLV-1)	Adult T-cell Leukemia/Lymphoma	Tax,	Constitutive splicing (113, 114)	Yes (115, 116)
		HBZ	Alternative 5’SS Intron retention (38)	Suggested (116)

The seven human cancer-inducing viruses are listed and classified here with their respective oncogenes and related cancers. If the said oncogenes are regulated by alternative splicing, the alternative splicing events (ASEs) in question are mentioned and references are found between brackets. Their role in splicing modulation is also precised. N/D = Not Documented.

## RNA splicing in HTLV-1 infection

Despite a growing interest in understanding the link between splicing mechanism and cancer onset and evolution, not much has been done concerning HTLV-1 and related ATL. However, Morteux’s team study from 2014 did provide a few elements of clarity on the matter. Using microarray comparative analysis, the authors showed that HTLV-1 infected but untransformed cells display a significant modification in alternative exons usage (AEU). Similarly, ATL (leukemic patients) cells exhibited the same behavior but at a higher level, as the authors calculated an 11-fold increase in AEU events (117). When looking at the >300 genes affected by those splicing events, mostly cancer pathway-related genes can be found. This leads us to wonder how HTLV-1-infected cells can use these alterations to their advantage, and if it plays a bigger role in the pathogenesis and ATL onset than just promoting proteome diversity and cell plasticity. As mentioned before, most HTLV-1 regulatory and auxiliary genes undergo splicing for mRNA maturation before translation into one unique protein (**Figure 1**)**;** but only *hbz* produces different protein isoforms. Splicing alterations in HTLV-1 could then slightly affect the good expression of viral genes, compared to genes involved in major cellular pathways. Hence, it is legitimate now to consider the role of HTLV-1 regulatory proteins in the modulation of alternative splicing pathways.

The role of Rex in HTLV-1 pathogenesis has been extensively reported already. Indeed, it is known for mediating the transport of unspliced or incompletely spliced viral structural proteins out of the nucleus to promote viral particle formation (118, 119). Although its potential role in splicing modulation had not been tackled in the HTLV-1 infection context yet, few studies did look into it, but in the HTLV-2 framework. Bakker et al. described decades ago that HTLV-2 Rex is a potent inhibitor of splicing *in vitro* and at an early step in spliceosomal assembly. Therefore, this inhibition of early spliceosome assembly by Rex may be responsible for the differential accumulation of unspliced transcripts, leading the latter to being transported out of the nucleus (120). With that in mind, we can consider that Rex could possess such splicing regulation function in the HTLV-1 context as well. In the remaining sections, we review current knowledge about Tax and HBZ’s potential role in splicing regulation and reflect on how dysregulation of this pathway, maybe by those two oncogenes, could participate in HTLV-1-induced cellular transformation and adult T-cell leukemia/lymphoma development.

### Splicing modulation by Tax

Regarding Tax and considering everything that was mentioned so far, it seems natural to question whether or not this viral transactivator and oncogene plays any role in the splicing mechanism regulation. Ben Ameur et al. were the first to tackle this particular aspect (115). Using RNA-sequencing analyses in reporter cells with a transient expression of Tax, the authors described that >900 genes were affected at the splicing level. Among all the splicing-altered genes recorded, only a minority of those were also altered at the expression level, meaning that Tax greatly impacts alternative splicing, independently from its transcription-mediation function. Half of the splicing modifications were also detected in ATL patients’ samples, confirming the previous results. Considering that no significant modification occurred in splicing factors-encoding genes upon Tax expression, the authors found that Tax-induced splicing alteration was dependent on NF-KB activation. In addition to the well-described functions of Tax on this major pathway (29, 121, 122), Tax activates NF-KB transcription factor RelA, which locally recruits the auxiliary spliceosome component DDX17. This splicing regulator then modulates splicing *via* its RNA helicase activity (115). This study constitutes the first step for deciphering an additional regulation role of Tax, this time in the splicing modulation of HTLV-1-infected cells.

Later on, the same team assessed the occurrence of different splicing events (see the 6 first events listed in **Figure 3A**) upon Tax expression (116). Results showed that Tax mostly promotes exon inclusion in their model; and that the splicing targets were enriched for cancer-related genes. Co-IP assays could confirm the interaction between Tax and U2AF65 (U2AF large sub-unit, see **Figure 2**) mentioned before, adding to the list of spliceosome-related interactants of Tax. Taken together, those two studies indicate a non-negligible role of Tax on alternative splicing regulation of mostly cancer-related genes, in a DDX17 and U2AF65-dependant manner (115, 116). These results contribute to enriching the diverse functions of Tax in HTLV-1-mediated pathogenesis and especially leukemogenesis.

### Insights regarding HBZ

As Tax is mainly expressed in the early stages of infection, or only transiently during the late ones, it seems necessary to question the role of the other HTLV-1 major viral oncogene on the splicing regulation matter. Unfortunately, this particular aspect was never approached, or only briefly. Indeed, Vandermeulen et al. also looked into the HBZ effect on splicing and uncovered that HBZ expression resulted in the occurrence of twice more splicing events compared to Tax (116). Authors noted opposite effects of Tax and HBZ, especially regarding exon inclusion and skipping (respectively) in their inducible Jurkat (immortalized T CD4^+^ lymphocytes) cells model. However, data obtained in ATL patients’ samples displayed a contrary result and indicated major exon inclusion promotion. As HBZ is the only viral gene expressed in all ATL cells, we hypothesize that the HBZ effect on splicing modulation is mainly resulting in this exon inclusion promotion type of event and that the study model may not be the most accurate for splicing study in an HTLV-1 infection context. Also, those observations could be confirmed in our experimental model using a bichromatic fluorescent reporter minigene in usHBZ or HBZ_SP1 stably-expressing cell lines. Y2H interactome revealed many HBZ-interactants related to RNA processing, especially splicing factors of interest such as PCBP1, SRSF2, or U2AF2, some overlapping with Tax. Again, analysis of the splicing targets revealed that HBZ mainly impacts cancer census genes indicating, in addition to proliferation and transformation functions previously cited. Hence, HBZ also seems to play a role in alternative splicing regulation; particularly of oncogenesis-associated genes.

## Discussion and future perspectives

Alternative pre-mRNA splicing is an essential mechanism that contributes to cellular plasticity and proteome diversity, giving cells an evolutionary advantage. It has now become clear that dysregulation of this key process greatly participates in transformation and maintenance of cancer cells. More and more works are contributing to deciphering the molecular mechanisms implicated in those aberrations, however, even with rapidly-advancing technologies such as high-throughput whole-genome and RNA-sequencing techniques, fully understanding the contribution of RNA splicing dysregulation in cancer biology is far from being achieved. In this review, we discussed the different levels of alternative splicing impairment of and by cellular and/or viral oncogenes and the consequences in the establishment of a cancer-prone environment. Given all the knowledge already acquired on the matter, it seems crucial to now consider this RNA splicing alteration aspect as a target in the development of future therapeutic approaches to either prevent, treat or even re-sensitize cancer cells to current chemotherapeutic strategies. Indeed, recent studies have highlighted the role of aberrant splicing in the occurrence of drug resistance mechanisms in many different cancers. Whether it contributes to chemoresistance by modifying the drug targets’ gene expression, or by diversifying the drug efflux-associated ones, alternative splicing can impair the global gene expression profile of a cancer cell, thus conferring survival benefits. The context of viral infections, adding to the already highly complex cancer framework, is even more poorly understood as it is the theater of an even greater number of splicing alterations. Considering the potential role of viral oncogenes in modulation of RNA splicing, studying their impact on key cellular pathways seems relevant to better evaluate the oncogenic and drug resistance properties they could confer to the infected cells. Regarding the particular case of HTLV-1 infection and related adult T-cell leukemia/lymphoma, if the molecular mechanisms of cell transformation and cancer onset and maintenance have been documented before, little is known about its oncogenes *tax* and *hbz* role in RNA splicing modulation. We reviewed here the current knowledge on the matter and stress the need of gaining more in-depth insights. As adult T-cell leukemia/lymphoma is a fatal malignancy for which no efficient treatment nor chemotherapies exist, exploring further the role of *tax* and *hbz* on alternative splicing regulation could give us the necessary tools to develop new approaches to treat this cancer.

## Author contributions

J-MP and JT conceptualized the article. Figures design and writing of the original draft and sections were carried out by JT. Reviews, correction and edition of the final version were done by J-MM and J-MP. All authors contributed to this review article and approved the submitted version.

## Funding

JT was supported by the University of Montpellier with a CBS2 doctoral school grant. J-MP was funded by the ARN 21-CO13-0002-01 and the SATT AXLR ISO-HBZ.

## Conflict of interest

The authors declare that the research was conducted in the absence of any commercial or financial relationships that could be construed as a potential conflict of interest.

## Publisher’s note

All claims expressed in this article are solely those of the authors and do not necessarily represent those of their affiliated organizations, or those of the publisher, the editors and the reviewers. Any product that may be evaluated in this article, or claim that may be made by its manufacturer, is not guaranteed or endorsed by the publisher.
